# Monitoring Highway Stability in Permafrost Regions with X-band Temporary Scatterers Stacking InSAR

**DOI:** 10.3390/s18061876

**Published:** 2018-06-08

**Authors:** Keren Dai, Guoxiang Liu, Zhenhong Li, Deying Ma, Xiaowen Wang, Bo Zhang, Jia Tang, Guangyu Li

**Affiliations:** 1 College of Earth Sciences, Chengdu University of Technology, Chengdu 610059, China; daikeren17@cdut.edu.cn; 2 State Key Laboratory of Geohazard Prevention and Geoenviroment Protection, Chengdu University of Technology, Chengdu 610059, China; 3 Department of Surveying and Geo-Informatics, Southwest Jiaotong University, Chengdu 610031, China; 200331010056@swpu.edu.cn (D.M.); insarwxw@gmail.com (X.W.); caijialun@my.swjtu.edu.cn (B.Z.); RSLGY@my.swjtu.edu.cn (G.L.); 4 COMET, School of Engineering, Newcastle University, Newcastle upon Tyne NE1 7RU, UK; zhenhong.li@newcastle.ac.uk; 5 School of Civil Engineering and Architecture, Southwest Petroleum University, Chengdu 610500, China; 6 Sichuan No. 1 Surveying and Mapping Engineering Institute, Chengdu 610100, China; yc@my.swjtu.edu.cn

**Keywords:** InSAR, temporary scatterers, permafrost region, highway subsidence, thermokarst, season frozen soil

## Abstract

Interferograms with short wavelength (e.g., X-band) are usually prone to temporal decorrelation in permafrost regions, leading to the unavailability of sufficient high-coherence interferograms for performing conventional time series InSAR analysis. This paper proposes the utilization of temporary scatterers for the stacking InSAR method, thus enabling extraction of subsidence in a permafrost region with limited SAR images and limited high-coherence interferograms. Such method is termed as the temporary scatterers stacking InSAR (TSS-InSAR). Taking the Gonghe-Yushu highway (about 30 km), part of G214 National Highway in Qinghai province (in a permafrost region), as a case study, this TSS-InSAR approach was demonstrated in detail and implemented. With 10 TerraSAR-X images acquired during the period from May 2015 to August 2015, the subsidence along this highway was extracted. In this case the lack of a consistent number of SAR acquisitions limits the possibility to perform other conventional time series InSAR analysis. The results show that the middle part of this highway is in the thermokarst and seasonal frozen soil area, and its accumulated subsidence reach up to 10 cm in 110 days. The thawing phenomena is still the main reason for the instability of highway. The results demonstrate that the TSS-InSAR method can effectively extract the subsidence information in a challenging scenario with limited X-band SAR images and limited high-coherence interferograms, where other time series InSAR-based techniques cannot be applied in a simple way.

## 1. Introduction

China is the third largest country in the world in terms of permafrost distribution. The proportion of permafrost and seasonal permafrost distribution accounted for 21.5% and 53.5% of the national land, respectively [1]. In the permafrost environment, a series of processes (e.g., freezing and thawing, swelling and contraction) alternately evolve, which cuase great potential harm to civil engineering facilities, agriculture and animal husbandry production. At the same time, the harm will be intensified as China has built the pavement with asphalt, in which the heat and the water are easy to be absorbed and sealed. These two factors make road construction and safe operation a challenge in permafrost regions. As a result, the instability of the highway subgrade in the permafrost environment of the Qinghai-Tibet Plateau has become a perennial problem for highway construction and maintenance in this area.

The subsidence monitoring of highway subgrade in permafrost environments is an important aspect for maintenance work and highway safety. At present, the commonly used subgrade subsidence monitoring methods are divided into two categories, one are the geotechnical measurement methods [2] (subsidence plate method, layered gauge), and the others are conventional geodetic methods [3] (such as GNSS, leveling). These two kinds of methods can effectively monitor the subsidence along the highway by point observation. Although high accuracy can be obtained for each single point, these two methods require a large number of field observations under extreme weather conditions, which makes it low-efficiency and high-cost. At the same time, these methods have some technical defects, i.e., the insufficient coverage for nearby areas and the low spatial resolution.

In recent years, spaceborne Synthetic Aperture Radar interferometry (InSAR), with its characteristics of high precision, high spatial resolution, weather-independent and wide coverage, has developed into an important technique to monitor surface displacements. It has its unique advantages in large-scale surface displacement monitoring. The time series InSAR technology (represented by PSI [4], SBAS [5] and others [6,7,8]) has been widely used in many areas such as high-speed rail settlement monitoring [9,10], urban subsidence monitoring [11,12,13,14,15] and landslide displacement monitoring [16,17,18,19,20,21]. A large number of studies have used time series InSAR to monitor surface deformation in permafrost regions with L-band (e.g., [22,23,24]) and C-band (e.g., [23,25,26,27,28]) images. In terms of X-band image use in permafrost regions, Wang et al. [29,30] studied the seasonal deformation features on the Qinghai-Tibet railway with a 1 km × 1 km study area and limited images. Short et al. [31] compared X-band, C-band and L-band images in a case study on Herscel Island, Canada, pointing out that with X-band images it is more difficult to detect long-term ground movement trends. Overall, successful cases of X-band use in permafrost regions are rare due to the low coherence from short wavelengths. It should be noted that in these conventional time series InSAR technologies, a large number of images is required for a reliable result. For example, in the PSI technique the PS points are selected based on their amplitude stability along the whole set of images therefore a minimum of 30 images is required for a reliable selection [32]. In the SBAS technique, enough available interferograms are needed to avoid separate SAR acquisition and to perform the SVD method [5].

In this paper, the InSAR technique is used to monitor the subsidence of the Gonghe-Yushu highway, part of the G214 national highway in Qinghai Province. Only 10 TerraSAR-X (TSX) SAR data covering study area were acquired from May 2015 to August 2015. In this permafrost area the interferometric coherence decreases sharply with the increment of the time interval due to the short wavelength of the TSX SAR images (i.e., X-band). The limited number of SAR images (i.e., only 10 TSX images) and insufficient high-coherence interferograms were the biggest challenges in this case. In such a challenging condition, the conventional time series InSAR-based techniques cannot be applied in a simple way. We monitored the highway stability by proposing a method called temporary scatterers stacking InSAR method (TSS-InSAR), which utilizes the temporary scatterers in the stacking InSAR method. The stacking process and proper selection of temporary scatterers can effectively overcome the limitations in this case study. The parameters and model of this method are discussed and analyzed in detail, in which we demonstrate how to control the different error source to use this method in a unique and challenging environment (e.g., reference 3D DEM was used to remove the phase error caused by residual terrain, data from weather stations was used to make sure of the removal of periodic displacements). With this method the subsidence along the highway is extracted and the related interpretation is performed with the geomorphology.

## 2. Study Area and Datasets

The Gonghe to Yushu highway, part of the G214 national highway (about 30 km), is the main research objective in this study. This highway is located in hinterland of the Qinghai-Tibet plateau with high altitude and very cold winters. There are up to seven months of frozen period throughout the year. Perennial frost exists in the high mountains and most of the rainfall is concentrated in May to September. The maximum temperature difference between day and night reaches 15 °C. This highway is in extremely poor natural conditions with high altitude and poor traffic conditions. It is cold and hypoxemic for people to live there, resulting in a sparse population. Therefore, it is costly and inefficient to perform conventional measurements there. This highway covers a wide permafrost area and the freezing and thawing phenomena are prominent, which brings great potential hazard to the highway subgrade. InSAR technology has its unique advantages in monitoring the subsidence in such area with wide coverage.

**Figure 1:**
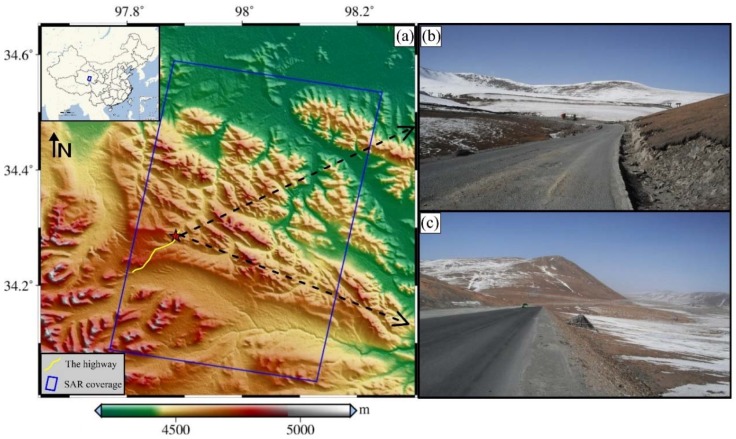
(**a**) The location of study area in China, the highway and the coverage of SAR image; (**b**,**c**) photos on site.

As the study area is located in a high altitude permafrost area, the surface would subside in the summer due to thawing and uplift in the winter due to freezing. Especially in the summer, due to the increase of temperature and rainfall, the subsidence phenomenon caused by permafrost thawing is obvious. In order to monitor the subgrade subsidence of this highway in the summer (during the thawing season), 10 TSX images were acquired from May 2015 to August 2015. The coverage of the SAR images is shown in Figure 1 with the blue frame, which is in Qinghai Province of China. The yellow line represents the Gonghe-Yushu highway, and on-site photos taken at the pentagram location are shown in Figure 1a,b. The altitude of the whole area is about 4000 m to 5500 m.

As the latest high-resolution SAR satellites from Germany, TSX data have a high resolution both in azimuth and range direction (i.e., 0.9 m range pixel spacing, 2.0 m azimuth pixel spacing), which can support the study for a specific highway. The TSX data has a short wavelength (3.1 cm), which is more sensitive to subsidence than other data with longer wavelengths. Even through the impact of decoherence can be reduced by its shortest revisit cycle of 11 days, it still has a significant influence on coherence of interferometry in this area. The dates of all the acquisitions and their related parameters are shown in Table 1.

## 3. Methodology

### 3.1. Temporary Scatterers Stacking InSAR Method

The stacking method is a simple and effective method in InSAR time series processing, in which a series of unwrapped interferograms are weighted and averaged according to the time span to estimate the average deformation velocity. It is mainly used to estimate the non-periodicity (approximate linear is best) average deformation velocity. The uniform surface displacements at mm/year accuracy can be extracted by the stacking method, which was confirmed by the validation with levelling data in some case studies [33]. Therefore, the results from the stacking InSAR method can be more accurate than conventional InSAR in some cases, while the other errors are preliminarily controlled (e.g., He et al. [34] used SRTM DEM and DELFT accurate orbit data to control the topographic errors, Fan et al. [35] analyzed the accuracy of SRTM DEM in the Tianjin area to control the topographic errors). This method have been recognized and applied by some researchers, e.g., [33,34,35,36,37].

As the interferograms in the permafrost region easily lose coherence, especially for the X-band SAR data, we combine the concept of temporarily coherent points with a stacking procedure to present a method called temporary scatterers stacking InSAR. Temporary scatterers (also known as temporarily coherent points [6], partially coherent targets [38], partially coherent pixels [39]) are the points that can maintain coherence during one or several intervals of SAR acquisitions. It is not necessary for them to maintain coherence during the whole time span.

**Figure 2:**
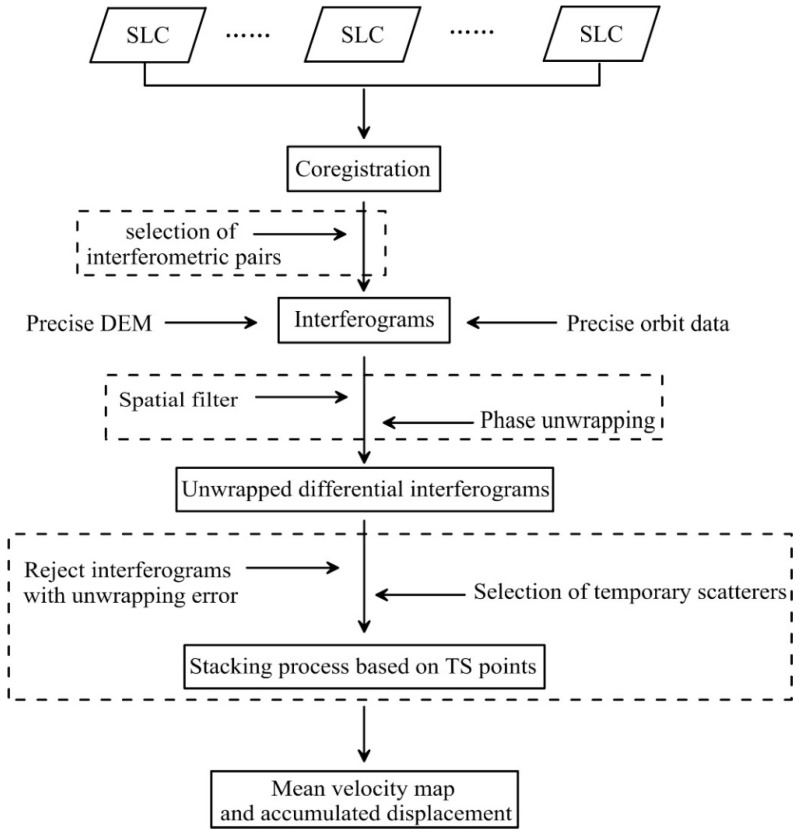
The flowchart of temporary scatterers stacking InSAR method.

The flowchart of this method is shown in Figure 2, and the details are as follows: after we got several SLC format SAR data covering the same area, co-registration was performed. A stringent temporal baseline and perpendicular baseline threshold were used to select the interferometric pairs with high coherence. The precise digital elevation model (DEM) and precise orbit data were used to remove the flattening effect and the topographic effect. After spatial filtering by the Goldstein filter [40], the temporary scatterers will be selected at this step, in which the correlation coefficient method [4] will be used.

Assuming *L* interferograms were generated by *N* + 1 SAR data, the correlation coefficient at a pixel can be calculated [41] within a selected *m* × *n* pixels (e.g., 5 *×* 5 used in this study) window by:

(1) $$\gamma_{l}=\frac{\left|\sum_{i=1}^{m}\sum_{j=1}^{n}M(i,j)⊗S(i,j)\right|}{\sqrt{\sum_{i=1}^{m}{\sum_{j=1}^{n}{\left|M(i,j)\right|}^{2}\sum_{i=1}^{m}\sum_{j=1}^{n}{\left|N(i,j)\right|}^{2}}^{}}},(l=1,2,\cdots ,L)$$

*M*(*i*,*j*), *S*(*i*,*j*) are the complex values on the pixel (*i*,*j*) at master and slave image, respectively. ⊗ denotes conjugate multiplication. *l* is the number of interferograms. After acquiring the correlation coefficient of an arbitrary pixel in the L interferograms, the threshold $\gamma_{crit}$ was set (e.g., 0.5 used in this study). If the following requirement was met, this pixel could be selected as temporary scatterer:

(2) $$C(\gamma_{l}>\gamma_{crit})>T_{},(l=1,2,\cdots ,L)$$

where *C*(·) denotes to count the variable, *T* is a threshold set before (*T* < *L*)*. l* is the number of interferograms. This method is a little different from the conventional correlation coefficient method. The conventional method sets the threshold for the minimum correlation coefficient, while this method sets the threshold for the minimum amount of pixels that exceed a correlation coefficient threshold. It is more suitable in this case as the interferograms that can be used are limited.

After temporary scatterers were selected, the spatial phase unwrapping was performed only on temporary scatterers to get unwrapped differential interferograms. The interferograms with obvious unwrapping error would be removed. The unwrapped differential phase at each temporary scatterers can be expressed as:

(3) $$\varphi_{diff}=\varphi_{def\_linear}+\varphi_{topo\_res}+\varphi_{atm}+\varphi_{noise}$$

$\varphi_{def\_linear}$ denotes the approximately linear displacement phase; $\varphi_{topo\_res}$ denotes the residue topographic phase; $\varphi_{atm}$ and $\varphi_{noise}$ denote the atmospheric phase and noise phase, respectively.

After removing or weakening the other phase component, the unwrapped differential interferometric phases consists mainly of the linear displacement phase, and they will be converted into a surface displacement by the following formula [42]:

(4) $$d=-\frac{\lambda }{4\pi }\varphi_{diff}$$

Finally, the average displacement rate and accumulated displacements could be calculated by the weight of time interval on each temporary scatterers point-by-point as the following formula:

(5) $$\bar{V}=\sum_{i=1}^{n}\Delta t_{i}d_{i}/\sum_{i=1}^{n}\Delta t_{i}^{2}$$

(6) $$\sigma (\bar{V)}\approx \sum_{i=1}^{n}{\left(d_{i}-\bar{V}\Delta t_{i}\right)}_{}^{2}/\Delta t_{i}^{2}$$

The interferograms with high coherence ensure the accuracy of phase unwrapping, and the introduction of the temporary scatterers ensure the stability and consistency of these point in time, which further ensure the accuracy and reliability of the final solution. Therefore, the concept of temporary scatterers in this paper is close to it used in other methods, such as the TCPInSAR method [6]. However, the proposed TSS-InSAR method is formally and substantially different from them. For instance, In the TCPInSAR method, offset was innovatively introduced as a criterion in TCP points identification [43], while in this study, we used correlation coefficient related factors to select points, i.e., Equation (2). In addition, to model the interferograms, the TCPInSAR method used a network concept to form the equations, in which the arcs were regarded as the basic observation [6], while in this study, we regarded the phase on points as basic observations.

### 3.2. The Feasibility of TSS-Insar Method in This Case

**Figure 3:**
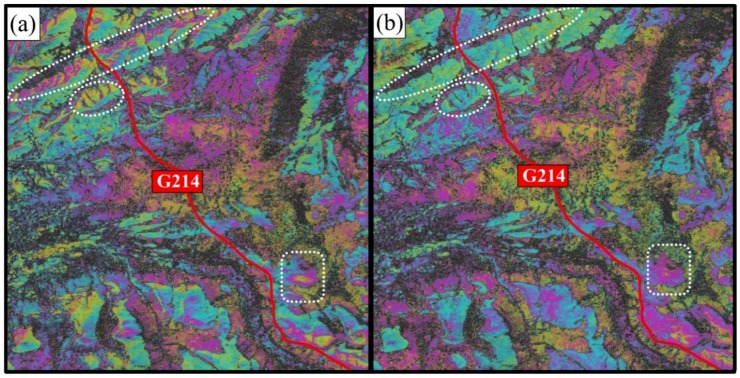
The comparison of interferograms after removing the topographic effect with (**a**) SRTM DEM; (**b**) Reference 3D DEM. The red line denotes the highway (based on SAR pixel coordinates).

In the stacking process, it is necessary to analyze each phase component and weaken the relative error source to ensure the accuracy of stacking. In terms of $\varphi_{topo\_res}$, after removing the topographic effect by SRTM DEM, it was found that there were obvious topography-dependent residual fringes in the interferograms (as shown in Figure 3a with a white circle), which means that in such a high altitude area, the vertical accuracy of SRTM DEM cannot meet our requirement to remove the topographic effect for InSAR. In this study, Reference 3D DEM was used to solve this problem. Reference 3D DEM is a high-accuracy DEM product from the French SPOT-5 satellite, which was generated by high resolution stereo (HRS) images. Based on the evaluation by other studies, the mean square error of its elevation reaches up to 1.95 m~3 m [44,45], which meet the needs of SAR interferometry [34,35,46,47]. By removing the topographic effect with Reference 3D DEM, the topography-dependent residue fringes disappeared as shown in Figure 3b in the white circle. The residue topographic phase can be effectively weaken by using this high-accuracy DEM.

**Figure 4:**
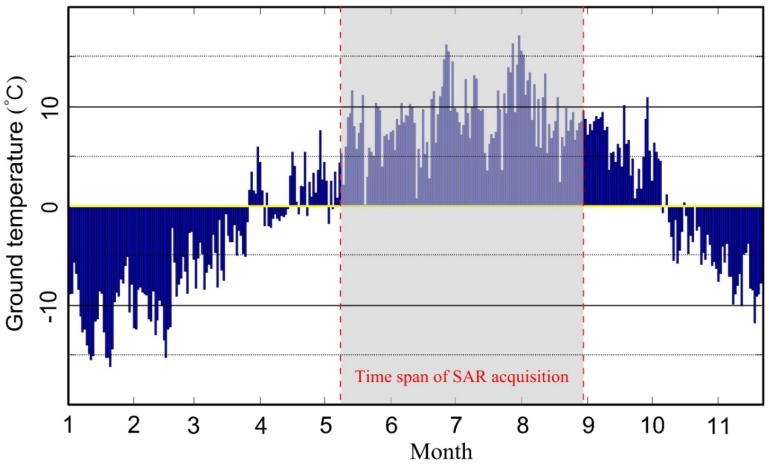
The daily average ground temperature in 2015 at the Qinghai weather station.

Considering that freezing and thawing are common phenomena in permafrost regions, it is necessary to consider the non-linear displacement phase or periodicity displacement in the differential phase. In the stacking method model, only linear displacements are modeled. In order to ensure there is no periodic displacement in the study area to perform the stacking method, we obtained the ground temperature data from January to November 2015 from the Qinghai Weather Station (as shown in Figure 4). It can be seen that from the end of April to the end of September the 0 cm ground temperatures were above zero, while the image acquisition period (gray area in Figure 4) was within this range. During this period, the frozen soil shows a tendency of thawing, indicating that the frozen soil will continuously subside instead of uplift by freezing. Therefore, with the consideration of the short time span of this period and the continuous thawing phenomena, the linear displacement model would be feasible and the uplift impact can be neglected.

To deal with the $\varphi_{atm}$ caused by atmospheric delay, the best way is the removal through external data, such as using GPS or MORIS data [48]. However, corresponding external data are not available for all regions. The stacking can effectively weaken the atmospheric influence based on the assumption that the atmospheric effect is relevant in space (around 1~2 km) and not relevant in time [34]. The $\varphi_{noise}$ was usually weakened by filtering. The selection of high coherence interferometric pairs can effectively make it negligible in this case. In addition, the proper selection of temporary scatterers can overcome some noise from phase unwrapping error. Based on the analysis above, the error source in the Equation (3) could be removed and weakened and TSS InSAR method was feasible in this case.

## 4. Results and Analysis

**Figure 5:**
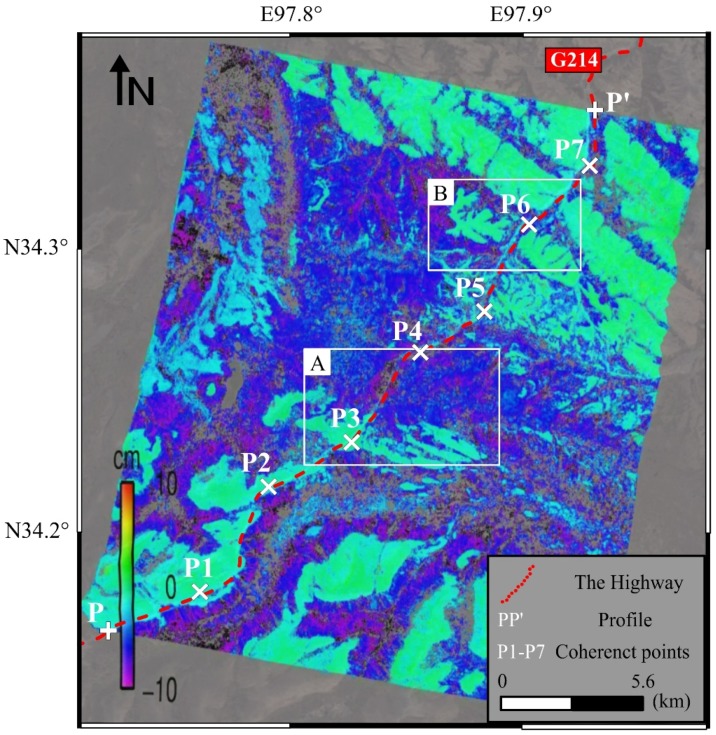
Accumulated subsidence map from stacking InSAR and the location of the highway, PP’, P1–P7.

By the temporary scatterers stacking InSAR method described above, the displacements along the Gonghe-Yushu highway (about 30 km) and its surroundings were monitored. The results are shown in Figure 5, in which the highway was represented by the red dashed line. Only the displacements on each selected temporal scatters were calculated and shown, and the remaining points were masked out (i.e., transparent points in Figure 5). Therefore, Figure 5 also shows the distribution of selected temporal scatters. The dense distribution indicates that the proposed method to select temporal scatters could dramatically increase the density of coherent points compared to only select the points maintaining coherence during the whole time span. The inhomogeneous subsidence along the highway can be found, with a maximum subsidence up to 10 cm in 110 days. Points P1–P7 were some coherent points that evenly distributed along the highway and their time series displacements are shown later. It should be noted that the monitoring time span was 110 days in total, which is just one third of a year. Therefore we presented the accumulated subsidence instead of the subsidence rate as usual.

In terms of spatial distribution, combined with the interpretation of optical images, the study area can be roughly divided into three types of geomorphy, i.e., permafrost area, seasonal frozen soil area and thermokarst area. Permafrost is ground, including rock or (cryptic) soil, at or below the freezing point of water 0 °C (32 °F) for two or more years [49]. Seasonal frozen soil refer to the soil that is frozen in winter and completely melted in summer. When the soil is frozen, the frozen soil has extremely high strength characteristics. While the soil melts, the soil will lose its strength completely and its physical and mechanical properties will change drastically with temperature [50], resulting in thawing-caused subgrade subsidence. The thawing-caused subgrade subsidence is one of the main hazards for infrastructure in frozen soil areas. In this region, the permafrost is distributed in blocks. As the altitude decreases, the seasonal frozen soil is split between permafrost and common land. Besides, there is a large area of thermokarst in the middle of this region. The spatial distribution of subsidence is highly correlated with the above geomorphic features. Therefore it can be simply inferred that geomorphy (soil type and so on) is the main factor that affects the inhomogeneous subsidence distribution in this area.

**Figure 6:**
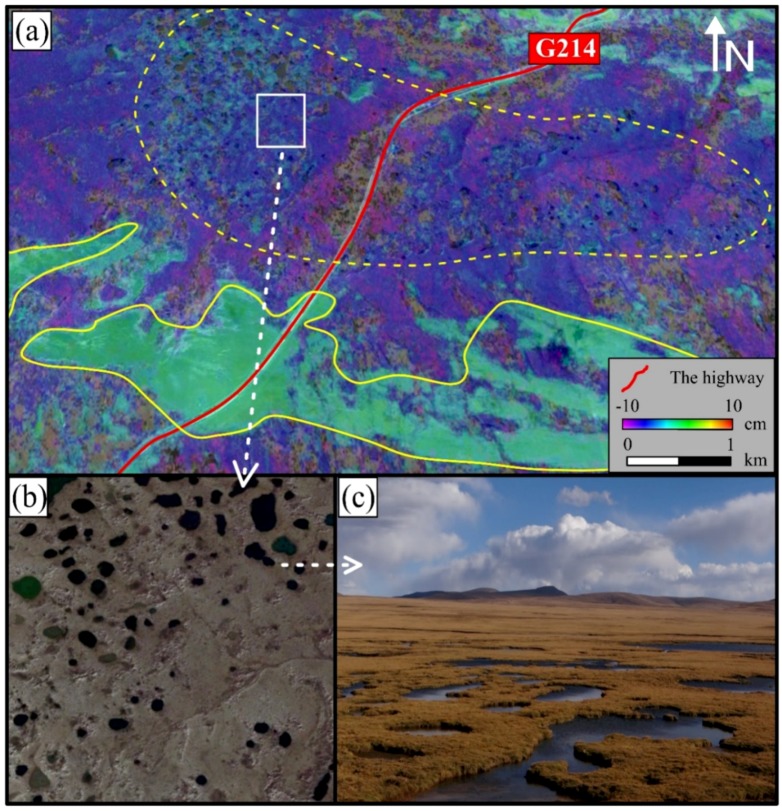
(**a**) The detailed subsidence distribution on region A; (**b**) High resolution optical satellite image from Google Earth; (**c**) Photos on-site from Google Earth.

It can be seen from Figure 5 that the maximum subsidence of the road occurred in the middle of the road (i.e., Area A in Figure 5 and shown in Figure 6). Figure 6b,c show the high-resolution satellite optical images and on-site photos of area A, respectively. In this area from south to north, the geomorphy changed from permafrost to soils which are prone to subsidence. The obvious water system and ponds in this area (in Figure 6a,b) are typical thermokarst ponds, which is one of the most notable features of permafrost degradation and is widely distributed in Qinghai-Tibet Plateau [51]. Based on this characteristics, large subsidence occurred of the highway going through this area, with an accumulative subsidence of 5 to 10 cm.

**Figure 7:**
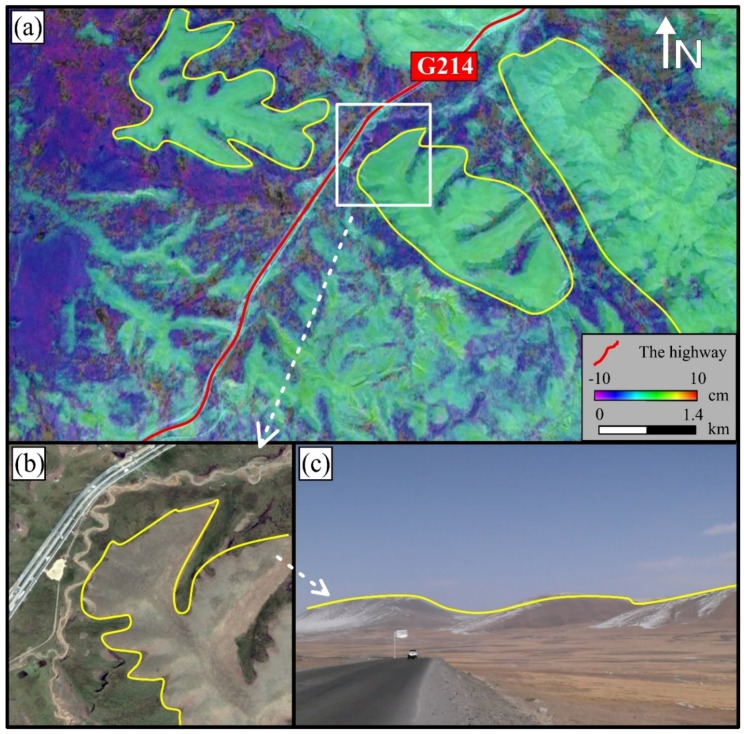
(**a**) The detailed subsidence distribution on region B; (**b**) High resolution optical satellite image from Google Earth; (**c**) Photos on-site from Google Earth.

After the area A, this highway entered a region with a combination of permafrost and seasonal frozen soil with a subsidence of 3 to 8 cm (Figure 7a). It can be seen that there is a clear difference between different soil types (gray and dark green in Figure 7a). The trend of subsidence is consistent with these soil types. On the snow-covered hills, the entire hill presents a green color for the subsidence (stable), while the subsidence increases gradually near the road. Based on the on-site photos, from the snow-covered hills to the road, the geomorphy changes from permafrost to seasonal frozen soil gradually, and the water content in the soil increases gradually, resulting in instability and large subsidence.

## 5. Discussion

### 5.1. Time Series Subsidence with Ground Temperature

**Figure 8:**
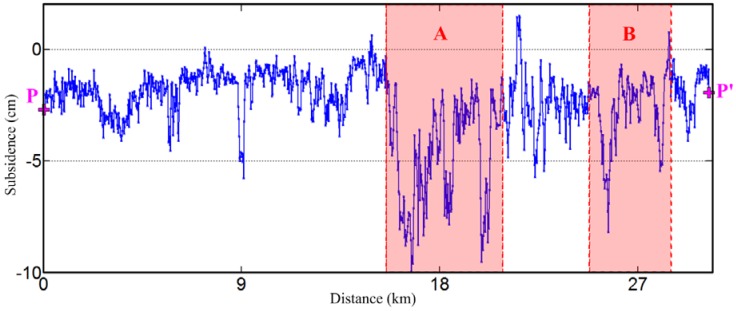
Subsidence profile along the highway, from point P to point P’ in Figure 5.

In terms of the subsidence along the highway, Figure 8 shows the profile of the Gonghe-Yushu highway (i.e., from point P to P’ in Figure 5). From west to east, this highway first passes through a stable area (about 0~15 km from point P) as can be seen from Figure 5. Although the adjacent area shows a relatively large subsidence, this part of highway was not greatly affected by the surrounding terrain and the subsidence is relatively stable, with a total subsidence of 5 cm. However, the central part has a thermokarst geomorphy with large subsidence on the highway and on both sides. The accumulated subsidence from May 2015 to the end of August 2015 (110 days in total) reached up to 10 cm. After that, the geomorphy was gradually changed into permafrost and the subsidence continued to decrease near to the point P’. In general, the overall average subsidence at the beginning and end of this highway was within 5 cm, which was acceptable and under control. Severe subsidence mainly occurred in the middle part of this highway (where there were many typical thermokarst ponds that can be seen in Figure 6b,c) with maximum subsidence approached 10 cm. There are two major subsidence areas (i.e., A and B area in Figure 5 and Figure 8), where long-term monitoring is necessary.

**Figure 9:**
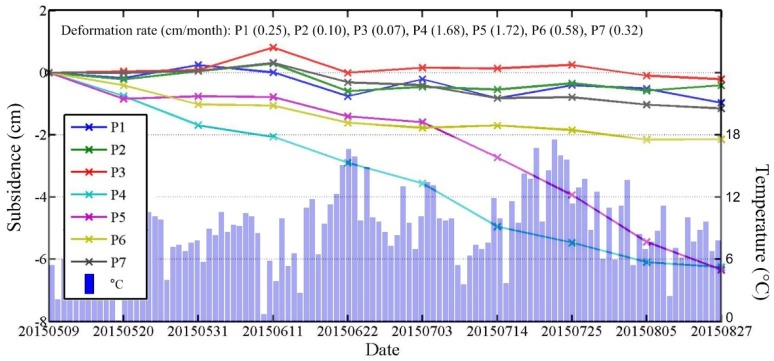
The time-series accumulated subsidence at point P1 to P7 with ground temperature data.

As temporary scatterers were used in this study, based on the definition as described in Section 3.1, the points that are able to maintain coherence during the entire time span (i.e., full-time coherent points) also would be selected in modeling and calculation. These points will help to study the time series subsidence of the highway. Seven full-time coherent points (P1 to P7 in Figure 5) which were roughly evenly distributed along the highway were extracted. Their time series subsidence together with the daily ground temperature are shown in Figure 9, where the horizontal axis represents the image acquisition date, and the left and right vertical axis represent the accumulated subsidence based on the initial time 20150509 and ground temperature, respectively. 

Points P1, P2, P3 and P7 were located in the stable area (i.e., the green region in Figure 5) with a maximum accumulated subsidence of 1.5 cm. Point P6 was located in a relative stable area, where the soil was near seasonal frozen soil with a accumulated subsidence of 2 cm. The points P4 and P5 were located in the thermokarst area in the middle part of this highway. Their maximum subsidence was more than 6 cm in 110 days. It should be noted that between P3 and P4 there was one part of the highway with the maximum subsidence that is close to 10 cm. We didn’t extract any full-time coherent points here, indicating that the big displacement was one reason for the loss of coherence. Combining the time series subsidence with ground temperature data, especially for points P4, P5 and P6, it can be seen that from the beginning of June, as the temperature increased the subsidence rate speeded up. From the middle of July to the beginning of August, the temperature reached up the highest value in one year, while the subsidence went into another accelerated period. After the beginning of August, the increment of subsidence rate decreased with the temperature decreased.

From the above analysis, it can be seen that the subsidence along the highway has a strong correlation with the nearby geomorphology. In the summer, the thawing of frozen soil contributes to the subsidence. In permafrost areas, the subsidence is small within a controllable subsidence rate. However, in the seasonal frozen soil area, the subsidence reached up to 5 to 8 cm during 110 days. Especially in the middle of the thermokarst area, the maximum accumulated subsidence was about 10 cm. The concerned departments should pay attention to these areas (e.g., A, B area in Figure 8) to prevent related disasters.

### 5.2. Possibility of Other Time Series Methods

**Figure 10:**
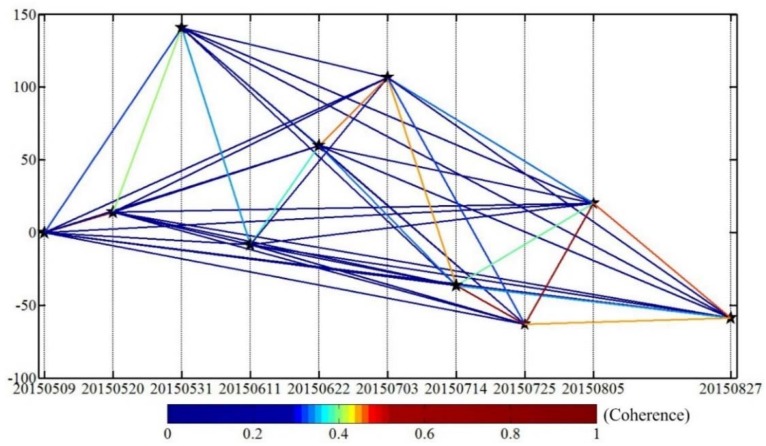
The temporal and perpendicular baselines of all the TSX interferometric pairs and their coherence (horizontal axis: the date of acquisition, vertical axis: the perpendicular baseline).

As we know, temporal variations in surface dielectric properties are common over permafrost areas due to changes in vegetation, soil moisture, and snow cover conditions [25]. Such variations can easily cause loss of coherence (or decorrelation) that corrupts interferometric signals [52], especially for X-band images. A coherence analysis was performed to check whether the conventional time series InSAR (e.g., PSI, SBAS) can be performed with the 10 X-band TSX images described above in this study. Figure 10 shows the spatial and temporal baseline of all interferometric pairs (45 pairs in total) formed by the 10 TSX images and their mean coherence coefficients. The overall coherence of all the interferograms is very low. For the interferograms with coherence coefficients lower than 0.3 (represented with a dark blue color), it is difficult to form continuous fringes, which would lead to incorrect phase unwrapping. The interferograms with coherence higher than 0.5 are regarded as high-quality ones to easily perform correct phase unwrapping (represented with a deep red color). Interferograms with coherence between 0.3 to 0.5 can be properly correctly unwrapped depending on the specific condition (represented by a gradually changing color). It can be seen that the interferograms in July and August have the best coherence. Only the interferograms with 11 days and 22 days intervals have the coherence above 0.3 (i.e., with color lines). All the interferograms with over 22 days interval have poor coherence (i.e., with dark blue lines).

**Figure 11:**
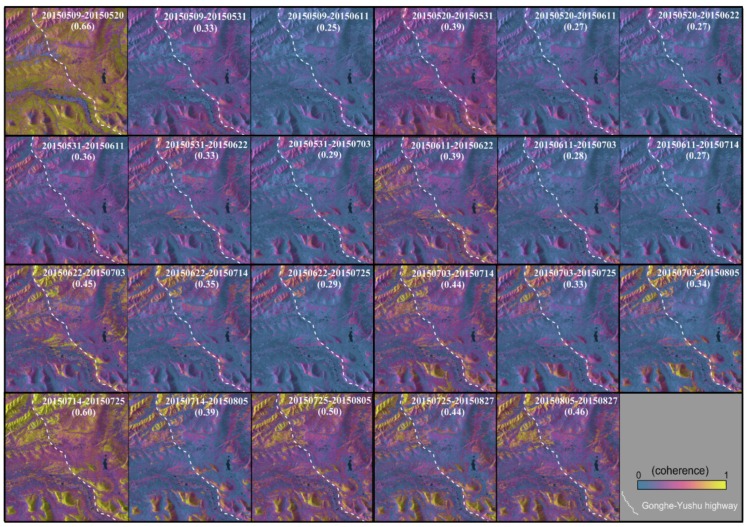
Coherence map of some interferometric pairs, imaginary line denote the G214 highway, (based on SAR pixel coordinate).

In addition, the coherence maps of all the interferograms with 11, 22 and 33 days temporal baselines are shown in Figure 11. The mean coherence coefficienta were shown in each subgraph within brackets. It can be inferred that the interferograms with 11 days temporal baseline can keep high coherence, while the coherence of interferograms with 22 days and 33 days dropped dramatically and hardly any useful information was extracted from them. Due to the short wavelength of the X band, a large number of interferograms lose coherence in this area and their coherence coefficients dropped dramatically with time.

From the above analysis we can infer that in this case the interferograms with large temporal baseline (i.e., >22 days) were decoherent. The correct phase unwrapping cannot be performed and useful information is hardly extracted from them. Therefore, we just use the interferograms with 11 days temporal baseline and the ones with coherence higher than 0.4, the number of which is less than 10. For the conventional time series InSAR methods (e.g., PSI, SBAS), this small number of interferograms are not enough to form a robust spatial-temporal network for calculation and successful subsidence measurement.

With the X-band, the phase signals are hard to maintain in correlation for robust differential phase measurements in permafrost regions. The successful use of the temporary scatterers stacking InSAR method in this study indicated that the conventional time series InSAR methods are not appropriate for all the cases. With limited images and limited high-coherence interferograms, some other methods, such as temporary scatterers stacking InSAR method, could be an alternative.

## 6. Conclusions

Based on X-band SAR images from the German TerraSAR-X satellite, this paper attempts to track the subsidence along the Gonghe-Yushu highway in a permafrost region. According to the coherence analysis, it was found that with short wavelength X-band, a large number of interferograms have very low coherence, leading to the unsuccessful implementation of conventional time series InSAR methods. This paper proposes the use of temporary scatterers for the stacking InSAR method, thus enabling extraction of the subsidence along this highway in a challenging scenario with limited SAR images and limited high-coherence interferograms. The basic idea, core processing steps, temporal scatterers selection method and phase model were introduced in detail. We discussed the feasibility of our method and the reference 3D high-resolution DEM data, ground temperature data from weather observation station were introduced to ensure the precision of the method performed in this case.

The results show that there is inhomogeneous subsidence distribution along this highway and the distribution has a strong correlation with the local geomorphy. The analysis of time series subsidence with ground temperature indicates that the thawing of frozen soil is the dominant factor for the subsidence in this area. There are two severe subsidence areas along the highway. The first part is in the middle thermokarst area, with a maximum accumulated subsidence of 10 cm in 110 days. The second part is located in the northeast part of this highway, where the subsidence of the seasonal frozen soil is 5 to 8 cm. For these two regions it is necessary to perform long-term monitoring and take measures to ensure the safety of the highway. This case study shows that the temporal scatterers stacking InSAR method could be applied in a challenging scenario with limited SAR images and limited high-coherence interferograms, where other time series InSAR-based techniques cannot be applied in a simple way, and it could be an alternative InSAR method for some challenging cases.

## Figures and Tables

**Table 1:** Dates of TSX acquisition and their related parameters.

No.	Date of Acquisition	Perpendicular Baseline (m)	Temporal Baseline (Days)
1	20150509	59	44
2	20150520	45	33
3	20150531	−80	22
4	20150611	68	11
5	20150622	0	0
6	20150703	46	11
7	20150714	−95	22
8	20150725	−122	33
9	20150805	−39	44
10	20150827	−118	66
